# Immunotherapy: A New Strategy for the Treatment of Cervical Cancer. Interview with Dr. Christian Marth and Dr. Sharad Ghamande

**DOI:** 10.3390/medsci6010009

**Published:** 2018-02-02

**Authors:** Medical Sciences Editorial Office

**Affiliations:** MDPI AG, St. Alban-Anlage 66, 4052 Basel, Switzerland; medsci@mdpi.com

## 1. Background

At the European Gynecological Oncology Congress (ESGO) 2017, held in Vienna, a symposium on immunotherapy took place: A New Strategy for the Treatment of Cervical Cancer, sponsored by Advaxis, Inc. 

Dr. Christian Marth, the chair of the symposium, and Dr. Sharad Ghamande, a Gynecological Oncologist coordinating a clinical study of the immunotherapy drug axalimogene filolisbac, shared with us their views about the current state of immunotherapy in cervical cancer. 

## 2. Short Biographies

Dr. Christian Marth is a professor in the Department of Obstetrics and Gynecology, Innsbruck Medical University, Austria, and the principal investigator for Advaxis’ trial to treat metastatic cervical cancer patients. He got his medical degree from Innsbruck University in 1985. From 1985 to 1991 he did his training in obstetrics and gynecology at the Department of Obstetrics and Gynecology at the Innsbruck University Hospital. In 1991, he obtained his PhD in obstetrics and gynecology.

**Figure:**
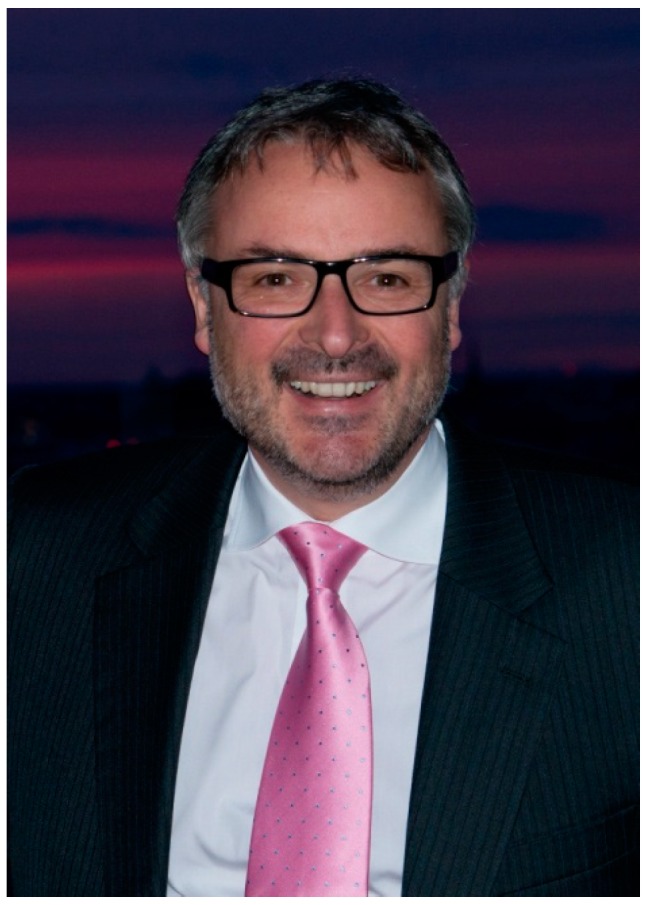
Dr. Christian Marth

Dr. Sharad Ghamande is currently Professor and Director of Gynecologic Oncology at the Georgia Cancer Center at Augusta University and a Principal Investigator for Advaxis. He is board-certified in both obstetrics and gynecology and gynecologic oncology. Dr. Ghamande completed his fellowship in gynecologic oncology at Roswell Park Cancer Institute in Buffalo, New York in 2000. His residency in obstetrics and gynecology was completed at Boston Medical Center in Boston, Massachusetts.

**Figure:**
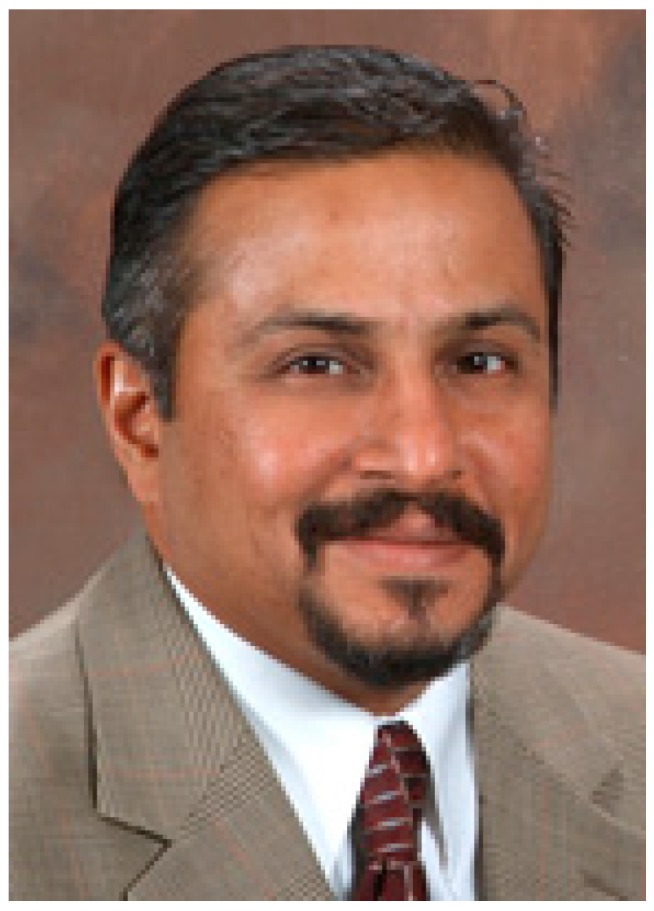
Dr. Sharad Ghamande

## 3. Interviews

### 3.1. Interview with Dr. Marth

**Q1. In recent years, we have heard more and more about immunotherapy. Can you explain what it is and how it can be used to treat cancer?**

R1. Immunotherapy is treatment that uses the immune system to fight diseases such as cancer. This can be done in different ways by stimulating the immune system to attack cancer cells. These cells have to be recognized as foreign and can then be killed by the immune cells. Different types of immunotherapy are used to treat cancer. They include:

- Checkpoint inhibitors (these are monoclonal antibodies that block cancer cells’ ability to inhibit the immune system) and
- Treatment vaccines (which work against cancer by boosting the immune system and inducing a response against cancer cells).

**Q2. As far as I know, there are experimental treatments based on immunotherapy for different types of cancers. How has the efficiency been so far?**

R2. Immunotherapy drugs are now used to treat many different types of cancer. For advanced or metastatic melanoma, bladder cancer, kidney cancer, and lung cancer, immunotherapy has resulted in an outstanding improvement in the survival of treated patients. For the first time, it can be speculated that a therapy is able to cure metastatic cancer patients. For some indications, immunotherapy is already standard care. For others, it is still experimental.

**Q3. Do you think immunotherapy can change cancer treatment in the short term?**

R3. Immunotherapy is one of the most important achievements in oncology. For many—maybe not all—types of cancer, immunotherapy, in the short term, will be included in the frontline therapy and has the potential to increase the cure rate.

**Q4. What is the state of the art of immunotherapy in gynecological cancers?**

R4. In gynecologic malignancies immunotherapy is still experimental, although very promising. For a special subtype of endometrial cancer with so-called mismatch repair deficiency, checkpoint inhibitors have already been approved by the FDA. In other indications, such as ovarian cancer or cervical cancer, trials are ongoing. We will start a very innovative trial soon by using a modified bacterium to bring tumor antigens into the patient’s body. These bacteria will be recognized by the immune system and, in an attempt to destroy them, tumors having the same antigens will be destroyed.

### 3.2. Interview with Dr. Ghamande

**Q1. Advaxis is one of the biotechnological companies working on the development of immunotherapy. Focusing on Advaxis’ axalimogene filolisbac immunotherapy, what does it consist of?**

R1. Axalimogene filolisbac (ADXS-11) is a unique immunotherapeutic vaccine that has been developed against HPV-related cancers, which include cervix, anal, and head and neck cancers. It uses the common cancer-causing strain HPV 16, E7 construct, which is fused with a common bacteria called *Listeria* that is inactivated and non-pathogenic. This combination is then injected intravenously and causes a targeted cytotoxic T cell immune response against HPV-bearing malignant cells. The *Listeria* also helps to influence the tumor micro-environment favorably by decreasing antagonistic cells like MDSCs and Tregs.

**Q2. The drug has been evaluated in a phase 2 study, and has been tested in a group of patients with persistent or recurrent metastatic carcinoma of the cervix. What are the characteristics of the study and what are its outcomes?**

R2. This drug has been studied in six clinical trials, either ongoing or completed. The pivotal trial was GOG 265, which had patients with recurrent, metastatic, or persistent cervical cancer who had failed at least one prior therapy and were treated with ADXS-11. The 12-month survival was 38% (a 52% improvement over historical controls), which was better than most of the phase 2 chemotherapy trials done in this patient population before. This treatment was safe and well tolerated, and a post-hoc analysis of the study has shown a potential new prognostic biomarker called AAT (Alpha Antitrypsin). These results mirror the data of a prior phase 2 study conducted in India with 110 patients who were randomized to ADXS-11 or a combination of the drug with five doses of weekly cisplatin. There was no difference in the response or survival in the two groups, with overall survival of 34.5% at 12 months and 24.8% at 18 months. https://ir.advaxis.com/press-releases/detail/1261/advaxis-presents-oral-late-breaking-data-on-phase-2.

**Q3. After the study, will it be cost-effective for both patients and the healthcare system?**

R3. Patients with recurrent cervical cancer have limited options, as this type of cancer is lethal and its treatment remains a huge unmet need. This is an exciting therapy that has the potential for prolonging survival in these patients. The most cost-effective way of using this therapy is perhaps in the adjuvant/first-line setting for women with advanced cervical cancer and those with nodal disease. Fifty percent of these patients will have a recurrence or die within four years. There is an ongoing phase 3 trial called AIM2CERV, which randomizes these patients 2:1 to the ADXS-11 versus a placebo for one year to prevent recurrence and impact long-term survival.

**Q4. What is your vision for the future regarding this specific drug and immunotherapy in general?**

R4. Most cervical cancers are caused by high-risk HPV, and have E6 and E7 oncogenes that are constitutively expressed, tumor-specific, and functionally important. That means cervical cancer can be attacked by different immunotherapy approaches more successfully than other gynecological cancers. We think that, along with ADXS-11, immunotherapy will play a big part in treating cervical cancer—mirroring the success of HPV vaccines in preventing cervical cancer.

